# Feasibility of Decentralised Deployment of Xpert MTB/RIF Test at Lower Level of Health System in India

**DOI:** 10.1371/journal.pone.0089301

**Published:** 2014-02-26

**Authors:** Neeraj Raizada, K. S. Sachdeva, Achuthan Sreenivas, Bhavin Vadera, R. S. Gupta, Malik Parmar, Shubhangi Kulsange, Ameet Babre, Rahul Thakur, Christen Gray, Ranjani Ramachandran, Umesh Alavadi, Mayank Ghedia, Balasangameshwara Vollepore, Puneet Dewan, Catharina Boehme, C. N. Paramsivan

**Affiliations:** 1 Foundation for Innovative New Diagnostics, New Delhi, India; 2 Central TB Division, Government of India, New Delhi, India; 3 World Health Organization, India Country Office, New Delhi, India; 4 Foundation for Innovative New Diagnostics, Geneva, Switzerland; California Department of Public Health, United States of America

## Abstract

**Background:**

Xpert MTB/RIF is an automated cartridge-based nucleic acid amplification test that has demonstrated its potential to detect tuberculosis and rifampicin resistance with high accuracy. To assist scale-up decisions in India, a feasibility assessment of Xpert MTB/RIF implementation was conducted within microscopy centres of 18 RNTCP TB units.

**Methods:**

As part of programme-based demonstration of Xpert MTB/RIF implementation, we recorded and analysed association between key implementation factors and the ability of test to produce valid results. Factors contributing to test failures were analysed from GeneXpert software data which provides ‘failure codes’ and causes for test failures.

**Results:**

From March’12 to January’13, total 40,035 suspects were tested by Xpert MTB/RIF, and 39,680 (99.1%) received valid results (Cumulative: 37157 (92.8%) on first attempt, 39410 (98.4%) on second attempt, 39637 (99.0%) on third attempt and 39680 (99.1%) on more attempts). Overall initial test failure was 2,878 (7.2% (4%–17%)); of these, 2,594 (90.1%) were re-tested and produced valid results. Most frequent reason of test failure was inadequate sample processing or equipment malfunction (3.9%). Other reasons included power failure (1.1%), cartridge integrity/component failure (0.8%), device-computer communication error (0.5%), and temperature-related errors (0.08%). Significant variation was observed in failure rates both across instruments and over time; furthermore, substantial variation was observed in failure rate in two cartridges lots.

**Conclusion:**

Installation required minimal infrastructure modifications and concerns about adequacy of human resources under public sector facilities and temperature extremes proved unfounded. Under routine conditions, Xpert MTB/RIF provided 99.1% valid results in TB suspects with low overall failure rates (7.2% initial failure, 0.9% final failure); devices provided valuable real-time feedback on reasons for test failure, which were used for rapid corrective action. High modular replacement (32%) and inter-lot cartridge performance variation remain sources of concern, and warrant close monitoring of failure rates as a key quality indicator.

## Introduction

Earlier and improved detection of all types of TB are global priorities for TB control. As conventional laboratory methods are time consuming, newer technologies for rapid detection remain as the focus of TB research and development. [1]

The WHO endorsed Xpert MTB/RIF (Cepheid, Sunnyvale, CA, USA) is a cartridge-based fully automated nucleic acid amplification test (NAAT) for TB case detection and rifampicin resistance detection, suitable for use in disease-endemic countries [2]. It extracts DNA, concentrates, amplifies, identifies targeted nucleic acid sequences in the TB genome, and provides results from unprocessed sputum samples in less than 2 hours with minimal hands-on technician time. [2]

The Xpert MTB/RIF test in principle enables diagnosis of TB and rifampicin-resistant TB at the clinics equipped with basic laboratory infrastructure supported by personnel with minimum technical skills. [3] Although testing with Xpert MTB/RIF does not require high standard laboratory set up, this sophisticated device requires careful handling. [4]

In controlled studies the Xpert MTB/RIF assay has demonstrated its potentials to detect tuberculosis and rifampicin-resistant TB with high sensitivity and specificity. [5] However, diagnostic tests performing well in controlled settings may not always perform optimally in settings of intended use. [6] Delivery systems have to account for several factors including specimen collection and transportation efficiency, device up-time, test reliability, environmental extremes, human resource constraints, reporting of results, supply chain, and multiple other critical factors beyond test accuracy. Therefore, before investing in scale-up, operational assessment of implementation should be conducted at the level of intended use. Accordingly, WHO has recommended country-specific operational research. [4]

In the present demonstration, we assessed operational feasibility of introducing Xpert MTB/RIF within the existing microscopy centers functioning under Revised National TB Control Programme (RNTCP) of India to inform decisions on scale-up of the technology under the programme. The objectives of the study were to collect evidence on the feasibility of implementation of Xpert MTB/RIF under routine conditions in existing microcopy centers; to assess test failure rates and the impact of key implementation factors on the assay in decentralized settings including the effect of variable temperature conditions, power failure, etc.; and to identify key issues that need to be monitored while implementing Xpert MTB/RIF test.

## Methods

**Figure 1:**
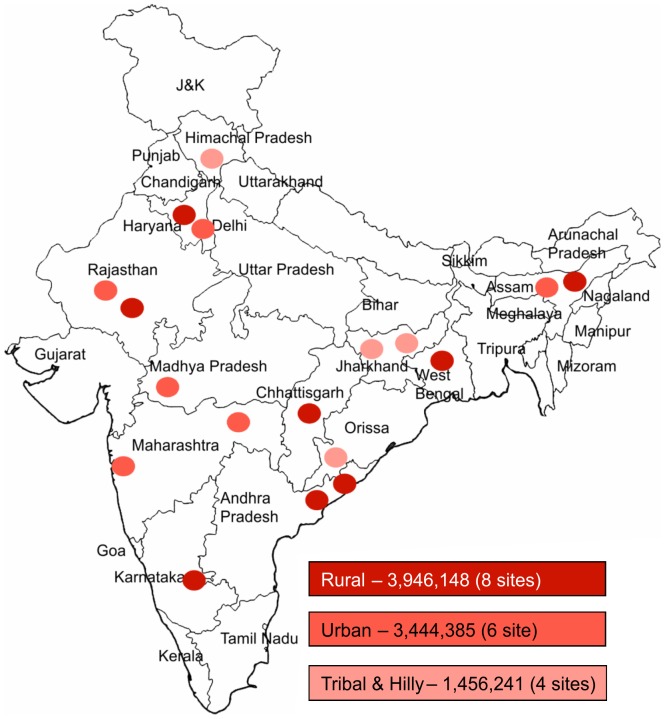
Site Locations: Geographical locations of the 18 project sites across India.

Study setting: The present demonstration was conducted in 18 selected RNTCP TB programme management units (TU) with an aggregate population of 8.8 million. Each TU caters on average to a population of 0.5 million, and encompasses 4–6 Designated Microscopy Centers (DMCs), and each DMC covers 2–4 health centres. Anyone attending these health facilities and suspected to have pulmonary TB are referred to a DMC for sputum smear microscopy. The 18 Xpert MTB/RIF study sites were selected by RNTCP to encompass the range of diverse geographic and demographic settings across the country, but restricted to those able to provide free MDR TB treatment services to those diagnosed for ethical purposes. 8 sites were in rural area, 6 sites were in urban area and 4 sites were in tribal and hilly areas covering a population of 3.9 million, 3.4 million and 1.4 million, respectively (Figure 1), and covering 99 DMCs and their linked health facilities.

Either one or two GeneXpert instruments equipped with 4 modules (GX–IV R2) were installed at all study sites. They were placed at the existing DMCs co-located within a selected TU by identifying a small room with electricity outlets, securing the space, adding air conditioner, and installing uninterrupted power supply units (UPS). Additionally, a cold room was required for cartridge storage. As ambient temperature beyond 30°C may affect assay performance, WHO guidance on rapid implementation of the Xpert MTB/RIF diagnostic test recommends temperature control. [4] A temperature gauge is preinstalled inside the GeneXpert instrument; when internal equipment temperature rises above the permissible limit, the equipment will not initiate an assay or will generate an ‘error’ result if there are assays underway. The UPS units were required to provide a minimum of 2 hours of power back-up, the maximum time for a single assay to complete. At two sites which were experiencing regular power failures, extended solar power back-up was provided during the study for the GeneXpert instruments.

Special arrangements were made for transportation of sputum samples from microscopy centres and public health facilities to the respective GeneXpert lab. Distance and travel time was assessed and a site-specific transportation mechanism was tailored to ensure that sputum samples reached the GeneXpert lab on the same day of collection even from more remote health facilities.

**Figure 2:**
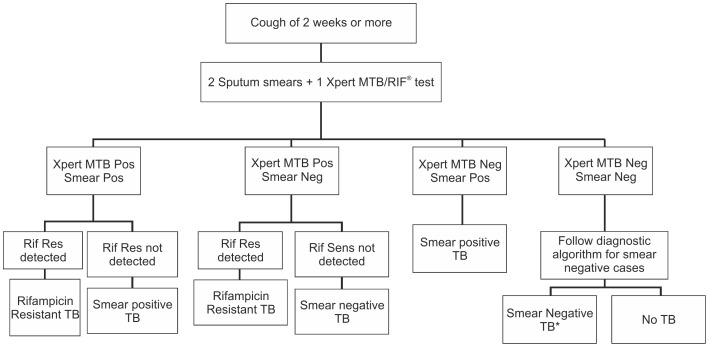
Diagnostic Algorithm.

A standardised algorithm (Figure 2) approved by the national technical committee for the project was used for the diagnosis of TB patients. All staffs were trained for uniform implementation of the algorithm across all sites; additionally, all were given hands-on training on the GeneXpert testing procedure as per manufacturer recommendations.

Feasibility assessment: The feasibility of Xpert MTB/RIF implementation was primarily assessed in terms of the ability of the assay to return a valid patient result. The absence of a valid test result for any given assay initiated was defined as a ‘test failure’ regardless of the underlying reason. Per patient, a test failure based on a single Xpert MTB/RIF test was defined as ‘initial test failure’; those initial test failures that could not be resolved on repeat assay, or which could not be re-tested for operational reasons, were defined as ‘final test failure’. We analysed the frequency of various reasons for the occurrence of test failure. In parallel, we routinely collected information on factors that may have affected failure rates, such as installation and training errors, operational non-availability, ambient temperature, power failure, equipment reliability, and cartridge manufacture lot.

The manufacturer has classified possible test failure causes as ‘error’, ‘invalid’ or ‘no result’. [7] An ‘error’ result indicates that the Xpert MTB/RIF assay in a given test was aborted by internal quality control mechanisms including improper filling of the cartridge reaction tube, cartridge reagent probe integrity failure, cartridge internal pressure excess, or equipment malfunction. All ‘error’ results are accompanied by specific error codes that provide additional information as to the underlying cause of failure. An ‘invalid’ result indicates that the polymerase chain reaction (PCR) has failed, usually due to the presence of PCR inhibitors. A ‘no result’ outcome indicates that the test underway was prematurely terminated either by external or internal factors during cartridge loading process, such as power failure, manual termination of the test by the operator, or one of the equipment or cartridge component failures. [7]

Under the study, for a patient, in case of ‘error’ or ‘no result’ outcome, repeat testing was performed on the same sample; for an “invalid” result, repeat testing was performed on a second fresh sputum sample due to concern over PCR inhibitors in the original specimen. The initial and final test failure rates were assessed across the study sites by directly extracting raw data from every test run initiated and recorded by the GeneXpert software.

Across 18 study sites, there were 27 GX–IV R2 instruments with 4 modules each for a total of 108 modules. Each module installed in a GeneXpert instrument functions independently from one another. A module failure leads to replacement of the specific module by the manufacturer if the equipment is under warranty or is covered under a maintenance contract. The frequency of such failures was assessed. At the time of analysis, error rates of prior 3 months were calculated. Modules with an error rate above 75^th^ percentile of error rate of all functional modules were defined as sub-optimally performing. Further analysis was conducted to assess trends in the performance of these modules over a period of time.

Similarly, to identify any possible variation between the performances of different lots of Xpert MTB/RIF cartridges, a lot-wise analysis of the invalid rate was undertaken as invalid results are generally considered to be independent of manual errors or other external factors affecting the overall test performance.

Ethics statement: The study protocol was approved by the Institution Ethics Committee of the National Tuberculosis Institute, Bangalore, India. Structured informed consent forms were used for obtaining written consent from all suspects enrolled in the study. Before taking consent, patients were informed about the study in vernacular language by the trained staff. For illiterate patients, after explaining in their mother tongue, the consent was taken in presence of literate witness. Approval for the study was granted by the Central TB Division, Ministry of Health and Family Welfare, Government of India.

## Results

Installation and training: The implementation of Xpert MTB/RIF testing began in 2012 at 3 demonstration sites in March 2012, scaled up quickly to 13 sites in May, and initiated at all 18 sites by August 2012. Xpert MTB/RIF assays were performed by the existing public sector laboratory technicians having no prior experience in molecular testing after a one-day structured hands-on training. Within 6 months of initiation an average enrolment of more than 5000 suspects per month was achieved across the project. The median number of tests conducted per working day per machine was 8, (IQR 5-12). No formal training sessions were required over the course of the project, though in instances where error codes more frequently indicated inappropriate sample processing, one-on-one re-sensitization on procedures were conducted.

Test failure rate: By the end of January 2013, a total of 42,971 Xpert MTB/RIF tests were conducted on 40,035 individual TB suspects (Table 1). A valid result was obtained for 37,157 (92.8%) on the first attempt. Out of the 2,878 initial test failures, repeat tests were done on 2,594 (90.1%), among which results were available from 2,523 (97.3%). Of the 2,594 TB suspects subjected to repeat testing, 2,253 specimen had valid results after a single repeat test and another 227 after a second repeat test (Table 2). Altogether, 39,680 (99.1%) of all TB suspects were provided valid results after repeat testing (Table 1, Table 2).

**Table 1:** Initial test results and final test results on Xpert MTB/RIF (N =  40,035 patients tested).

Test Results	No. of initial test results	%	Range across sites	No. of final test results	%	Range across sites
**Valid**	37157	92.8%	85.8% – 96.1%	39680	99.1%	96.5% – 100%
**Failure** *	2878	7.2%	3.9% – 14.2%	355	0.9%	0.04% - 3.5%
**Error**	1790	4.5%	2.6% – 11.2%	177	0.4%	0% – 2.7%
**Invalid**	459	1.1%	0.2% – 2.6%	142	0.4%	0% –1.5%
**No Result**	629	1.6%	0% – 3.9%	36	0.1%	0% –0.5%

* *Break up of test failure results - Error, Invalid, No results*

**Table 2:** Number of tests conducted per patient on Xpert MTB/RIF far a valid result (N =  40,035 patients tested).

Number of tests per patient	Number of patient specimen tested	Patients with valid result	%	Test failures	Test failures retested	%	Test failures not repeated	%	Cumulative number of valid results	%
Single test	40035	37157	92.8%	2878	2594	90.1%	284	9.9%	37157	92.8%
Single repeat test	2594	2253	86.9%	341	287	84.2%	54	15.8%	39410	98.4%
Repeat tests twice	287	227	79.1%	60	44	73.3%	16	26.7%	39637	99.0%
3 or more repeat tests	60	43	71.7%	17	16	94.1%	1	5.9%	39680	99.1%

The initial test failure rate was 7.2%, with a range of 3.9% to 14.2% across all 18 sites (Table 1, Table 2). Initial test failures were mostly classified as ‘errors’ [1,790 (4.5%)], with fewer ‘no results’ [629 (1.6%)] or ‘invalid’ results [459 (1.1%)] (Table 1).

Most of the repeat tests were also conducted on the same specimen and on the same day, without inconvenience to patients. Of the 2,594 TB suspects subjected to repeat testing, 1,998 repeat testing were performed on the same specimen. 1,961 (98.1%) of these were resolved. The other 596 were tested on a different specimen of which 562 (94.3%) were resolved with a valid test result.

**Figure 3:**
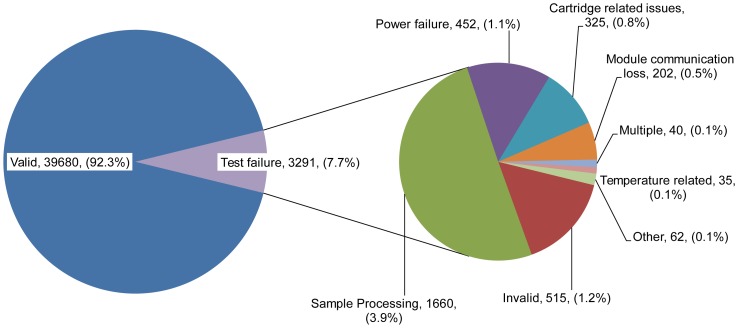
Reasons for test failure.

Test failure cause analysis: As per the test failure codes generated by the Xpert MTB/RIF, leading cause of test failure was sample processing error and equipment malfunction, contributing to a total of 1660 of 3291 test failures. The frequency of various factors contributing to test failures is described in Figure 3 and Table 3. Of these 1660 failures, more than 75% (1260) occurred at 9 sites. Similarly, more than 80% of the 515 invalid test results occurred across 9 sites (Table 2).

**Table 3:** Site wise distribution of various factors contributing the Xpert MTB/Rif test failures.

Site	Site A	Site B	Site C	Site D	Site E	Site F	Site G	Site H	Site I	Site J	Site K	Site L	Site M	Site N	Site O	Site P	SiteQ	Site R	TOTAL
Site profile	Rural	Urban	Urban	Urban	Urban	Rural	Rural	Urban	Tribal	Rural	Tribal	Urban	Urban	Rural	Hilly	Rural	Tribal	Rural	
Grand Total	5303	4741	4256	3737	3300	3177	3142	2917	2505	2049	1453	1361	1218	1150	801	751	679	431	42971 (92%)
Valid	4744 (89%)	4495 (95%)	4027 (95%)	3468 (93%)	3081 (93%)	2997 (94%)	2883 (92%)	2612 (90%)	2320 (93%)	1966 (96%)	1367 (94%)	1134 (83%)	1101 (90%)	1060 (92%)	691 (86%)	716 (95%)	614 (90%)	404 (94%)	39680 (4%)
Sample processing & Equipment related issues	217 (4%)	105 (2%)	151 (4%)	131 (4%)	98 (3%)	72 (2%)	118 (4%)	176 (6%)	71 (3%)	38 (2%)	69 (5%)	188 (14%)	76 (6%)	46 (4%)	51 (6%)	16 (2%)	25 (4%)	12 (3%)	1660 (1%)
Invalid	107 (2%)	13 (0%)	26 (1%)	17 (0%)	47 (3%)	59 (2%)	79 (3%)	26 (1%)	6 (0%)	6 (0%)	8 (1%)	31 (2%)	19 (2%)	23 (2%)	29 (4%)	13 (2%)	2 (0%)	4 (1%)	515 (1%)
Power failure	113 (2%)	28 (1%)	4 (0%)	60 (2%)	7 (0%)	24 (1%)	36 (1%)	44 (2%)	70 (3%)	0 (0%)	9 (1%)	4 (0%)	8 (1%)	9 (1%)	20 (2%)	0 (0%)	12 (2%)	4 (1%)	452 (1%)
Cartridge related issue	34 (1%)	82 (2%)	42 (1%)	10 (0%)	31 (1%)	10 (0%)	8 (0%)	42 (1%)	19 (1%)	13 (1%)	0 (0%)	0 (0%)	6 (0%)	6 (1%)	6 (1%)	2 (0%)	10 (1%)	4 (1%)	325 (0%)
Module communication loss	73 (1%)	10 (0%)	2 (0%)	39 (1%)	33 (1%)	4 (0%)	9 (0%)	7 (0%)	0 (0%)	20 (1%)	0 (0%)	0 (0%)	0 (0%)	0 (0%)	0 (0%)	0 (0%)	3 (0%)	2 (0%)	202 (0%)
Other	8 (0%)	6 (0%)	4 (0%)	2 (0%)	1 (0%)	5 (0%)	2 (0%)	3 (0%)	4 (0%)	5 (0%)	0 (0%)	4 (0%)	7 (1%)	4 (0%)	3 (0%)	0 (0%)	3 (0%)	1 (0%)	62 (0%)
Multiple	0 (0%)	1 (1%)	0 (0%)	7 (0%)	0 (0%)	3 (0%)	8 (0%)	0 (0%)	12 (0%)	0 (0%)	0 (0%)	0 (0%)	0 (0%)	1 (0%)	1 (0%)	0 (0%)	7 (1%)	0 (0%)	40 (0%)
Temperature related	2 (0%)	1 (1%)	0 (0%)	6 (0%)	2 (0%)	3 (0%)	0 (0%)	7 (0%)	4 (0%)	1 (0%)	0 (0%)	0 (0%)	1 (0%)	1 (0%)	0 (0%)	4 (0%)	3 (0%)	0 (0%)	35 (0%)
Total test failure	554 (10%)	246 (5%)	229 (5%)	272 (7%)	219 (7%)	180 (6%)	260 (8%)	305 (10%)	186 (7%)	83 (4%)	86 (6%)	227 (17%)	117 (10%)	90 (8%)	110 (14%)	35 (5%)	65 (10%)	27 (6%)	3291 (8%)

452 (1.1%) assays generated errors due to power supply related issues. 5 sites accounted for a bulk of such errors, 328 (72%). At two of these sites, as an intervention, solar power backup was provided which brought down the power-outage related error rate from 2.1% (131/6381) to 0.9% (18/2064) (p = <0.001, Pearson's Chi-Square Test).

A total of 35 (0.08%) test failures were associated with temperature related issues. These test failures were reported from 11 out of 18 project sites and 77.1% (27) of the 35 temperature related test failures occurred at 6 sites. These test failures were compared with the maximum and minimum ambient temperature recorded in the lab on the particular day when test failure occurred. This comparison showed that majority of these failures, i.e. 30 (85.7%), were caused by inadequate exhaust of warm air from the equipment and only 5 (14.3%) could be attributed to high ambient temperature.

Other causes for test failures were individual cartridge-related failures such as internal cartridge integrity test failure, cartridge syringe stall, cartridge component failure, etc. (325, 0.8%). Finally, loss of communication between modules and computer system accounted for 202 (0.5%) errors; however, 81.7% of these communication errors occurred at only 4 sites. (Figure 3, Table 3).

No trends on the basis of regional distribution, demographic profile or level of decentralisation of these sites were found in the factors contributing to the occurrence of test failures (Table 3).

Cartridge lot and module analysis: Stratification of test results by manufacturing lots showed that two particular cartridge lots were associated with a much higher frequency of test failures, due mostly to ‘invalid’ results. Of the 19 cartridge lots used 17 had median invalid rates of 0.5% (range 0.0 to 0.7%) whereas two lots had significantly higher invalid rates of 2.9% (14/487) and 5.3% (364/6869). Because cartridge-related test failures were immediately signalled to the device operators, most patients tested by this particular cartridge lot were re-tested and provided test results.

**Figure 4:**
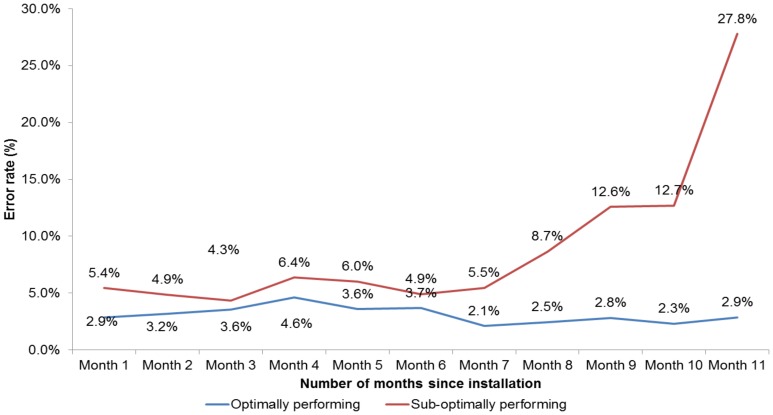
Trend in error rates of modules.

Of the total 108 modules in the study, 34 required replacements on account of various performance related issues; 10 had an overt malfunction and 24 showed degraded performance in test validity rates indicating impending malfunction. Of the 10 replaced modules, 7 modules had less than three months of data and were excluded from analysis due to a low comparative number of tests done (average 68 tests per module). 24 modules were identified with sub-optimal performance on account of higher test error rate as compared to the other modules. The overall test error rate in these 24 modules was 6.1%, while the overall test error rate in rest of 73 modules was 3.3% (p<0.001). It was observed that performance of these 24 modules had deteriorated over a period of time since installation (Figure 4). These 24 modules were later replaced by the manufacturer.

## Discussion

This multi-centre demonstration has shown the striking feasibility and ease of Xpert MTB/RIF implementation across a diverse range of settings, utilizing the existing human resources and infrastructure found in public sector microscopy centres. Real-time feedback on reasons for test errors directly from the devices allowed for easy and rapid detection of implementation gaps and led to quick correction of most causes. Concerns about the adequacy of public sector human resources, facilities, and temperature extremes proved unfounded. Staff required minimal training, and the low frequency of sample processing errors attested to the effectiveness of this minimalistic approach to implementation. Installation required only minor infrastructure modifications and provision of power backup, and areas with particularly poor power supply were well-served by low-cost solar power battery recharging, as demonstrated by the low frequency of test interruptions. With the routine addition of room air conditioners, temperature-related errors were quite rare.

Implementation of Xpert MTB/RIF resulted in a very high percentage (99.1%) of suspects being provided with valid results. Other studies conducted on Xpert MTB/RIF assay elsewhere have also documented similar high proportion of interpretable results. [5]–[6] [8] A very large implementation programme of Xpert MTB/RIF by the National TB Programme of South Africa has reported 97.3% interpretable results on a large sample size. [9] These findings highlight the usefulness of monitoring initial and final test failure outcomes and repeat testing of all initial test failures in ensuring a high proportion of valid results.

The proportion of interpretable results after retesting on Xpert MTB/RIF was significantly higher than other diagnostic tools recommended by WHO for detection of rifampicin resistant TB cases. A key factor contributing and crucial to the high proportion of interpretable results under the study was the feasibility of rapid retesting and thereby resolving a significant proportion of test failures on the same specimen, in absence of which proportion of interpretable results would have been significantly less. Studies conducted with MTBDR*plus* line probe assays for the diagnosis of DR-TB reported 92%–97% interpretable results. [10]–[14] Although liquid culture and DST systems for diagnosis provide a higher sensitivity and specificity, they are known to be more prone to contamination. Even in experienced laboratories, approximately 5–10% of specimens cannot yield results because of contamination. [15]–[16] Similarly, for the laboratory diagnosis of sputum smear negative TB, Xpert MTB/RIF with the advantages of a quick turn-around time, yield of a high proportion of reportable results and feasibility of rapid roll-out, provides a very promising alternative to solid and liquid culture testing for the diagnosis. [17]

The leading cause of test failure results observed in the present study was due to inadequate sample processing and equipment malfunction. Similar findings were documented by the early implementers of Xpert MTB/RIF assay. [18]–[19] Majority of the test failures on account of inadequate sputum processing were attributed to high viscosity of a small proportion of sputum samples. This finding suggests that to decrease such test failures, the incubation period of sputum in the sample processing buffer may be allowed for an additional 10 minutes for specimen with high viscosity [20].

Like any other automated laboratory technology, Xpert MTB/RIF requires a stable electric power supply, and even short term interruption of power would result in test failures. [4] In the present study, 1.1% initial test failures were attributed to power failure as some of the sites had prolonged power outage. The feasibility of effectively addressing power related issues with interventions such UPSs and solar power back up was demonstrated adequately in the present study.

The manufacturer recommends a maximum of 30°C ambient operating temperature for the operation of GeneXpert instrument and data on the robustness of the device under prolonged periods of temperature exceeding 30°C are not available. [17] Our experience with over 10 months at sites situated at predominantly warm settings, established that the temperature related concerns in Xpert MTB/RIF testing could be very effectively addressed with the installation of air conditioning units. Even during frequent power failures at the study sites leading to absence of air-conditioning, the temperature was usually maintained within the desired range and overall temperature related errors in our study were rare. It was further observed that majority of temperature associated errors was related to inadequate exhaust of warm air from the equipment either due to clogging of exhaust fan filter due to dust or inappropriate positioning of equipment. Based on our observation we recommend regular cleaning of fan filter along with appropriate positioning of the instrument to allow free flow of air to minimize the frequency of temperature related errors.

In the study, significant variation in the performance of GeneXpert modules and Xpert MTB/RIF cartridges was observed. Cartridges from two lots identified as associated with high failure rates were returned to the manufacturer for further investigation. However, it needs to be noted that this observation in one of the two lots was based on very small sample of tests and may need to be validated on a larger sample. The most striking finding, however, was that this could be rapidly detected – indeed this failure code analysis provides robust rapid data for ongoing automated quality assurance. The implementation of networked, real-time central monitoring software is expected to further aide early detection of potentially problematic lots or modules, if and when such problems arise.

While the performance of some modules deteriorated with time irrespective of the workload, performance of other modules continued to be satisfactory during the study period. Early implementation of GeneXpert technology in programmatic settings in Democratic Republic of Congo reported 39% module replacements due to technical reasons including unexplained high error rates in less than one year of use. [21] These findings warrant regular monitoring of inter-modular and inter-lot variation in performance of the Xpert MTB/RIF assay and any deviation in the performance need to be reported immediately to the manufacturer.

Overall it was observed that GeneXpert has the prerequisite robustness for rapid scale up at decentralised settings while maintaining quality of results. We were able to effectively address concerns related to ambient temperatures in warm climatic settings and erratic power supply. Further the study identified some aspects related to Xpert MTB/RIF testing such as initial and final test failure rate, inter-modular variation in performance over time, inter-lot variation in performance, etc. that may need to be monitored nationally, while rolling out this technology and deploying large number of systems.

Replacement of dysfunctional modules was possible as all the equipments were either under warranty or annual maintenance contract with the manufacturer, highlighting the importance of having such systems/services in place.

## Conclusion

This multi-centre demonstration has shown striking feasibility and ease of Xpert MTB/RIF implementation across a diverse range of settings, utilizing the existing human resources and infrastructure found in public sector microscopy centres. Installation required only minimal infrastructure modifications of air-conditioning and power back-up plus low-cost solar power battery charges in areas with particularly poor power supply. Concerns about the adequacy of public sector human resources, facilities, and temperature extremes proved unfounded.

Under routine conditions in public sector RNTCP microscopy centres, Xpert MTB/RIF routine testing of patients suspected of TB provided valid results in 99.1% of TB suspects tested with an overall low failure rates (7.2% initial failure rate (4–17%), 0.9% final failure rate).Devices provided valuable real-time feedback on reasons for test failure, which were used for rapid corrective action. Module reliability (32% required replacement ) and inter-lot variation of cartridges remain sources of concern, and warrant close central monitoring of failure rates as an indicator of potential quality problems. Further, in depth analysis of cost effectiveness of this device in such settings would help in decision making at national level.
